# Predicting language treatment response in bilingual aphasia using neural network-based patient models

**DOI:** 10.1038/s41598-021-89443-6

**Published:** 2021-05-18

**Authors:** Uli Grasemann, Claudia Peñaloza, Maria Dekhtyar, Risto Miikkulainen, Swathi Kiran

**Affiliations:** 1 Department of Computer Science, The University of Texas at Austin, Austin, TX 78712 USA; 2 Department of Speech, Language and Hearing Sciences, Boston University, Boston, MA 02215 USA

**Keywords:** Neurology, Computational models

## Abstract

Predicting language therapy outcomes in bilinguals with aphasia (BWA) remains challenging due to the multiple pre- and poststroke factors that determine the deficits and recovery of their two languages. Computational models that simulate language impairment and treatment outcomes in BWA can help predict therapy response and identify the optimal language for treatment. Here we used the BiLex computational model to simulate the behavioral profile of language deficits and treatment response of a retrospective sample of 13 Spanish-English BWA who received therapy in one of their languages. Specifically, we simulated their prestroke naming ability and poststroke naming impairment in each language, and their treatment response in the treated and the untreated language. BiLex predicted treatment effects accurately and robustly in the treated language and captured different degrees of cross-language generalization in the untreated language in BWA. Our cross-validation approach further demonstrated that BiLex generalizes to predict treatment response for patients whose data were not used in model training. These findings support the potential of BiLex to predict therapy outcomes for BWA and suggest that computational modeling may be helpful to guide individually tailored rehabilitation plans for this population.

## Introduction

Aphasia is the most common speech and language disorder that affects the bilingual adult population seeking language rehabilitation after brain injury (e.g., stroke). Bilinguals with aphasia (BWA) frequently present with varying degrees and patterns of impairment and recovery in their two languages^1,2^, which reflects the complex interplay between their unique prestroke bilingual experience and the effects of neurological damage to their bilingual language processing system. While there is evidence that treatment in either language can induce recovery^3^, most individuals show therapy effects in the treated language, while others may also show varying degrees of cross-language generalization in the untreated language^4,5^. These valuable findings, however, result from single case reports and studies with small heterogeneous samples, making it difficult to predict reliably the extent to which treatment gains will be observed across languages in BWA. Hence, identifying the optimal language for treatment, i.e., the one that leads to maximum therapy gains across both languages, remains an essential open question for bilingual aphasia rehabilitation research^6^, and one that has vast clinical service delivery implications. Importantly, the large individual variation in bilingual experience and post-stroke impairment in BWA poses two critical methodological challenges for behavioral studies: (i) how to include enough individuals with similar linguistic backgrounds and deficits so that factors that influence rehabilitation outcomes can be examined systematically, and (ii) how to generalize the findings to broader populations of BWA.

Because large-scale behavioral clinical studies are often unfeasible, computational modeling can provide an alternative method to predict the optimal language of treatment by simulating therapy outcomes in both languages, while accounting for both individual history of bilingualism and impairment in each language. Computational modeling can help characterize crucial mechanisms and relevant factors that underlie language acquisition, representation and processing^7,8^, and disentangle the role of variables that often become confounded in behavioral research^9^. Such modeling has been used to examine multiple aspects of language learning and processing in healthy bilinguals^10–17^ as well as a wide range of language processing deficits in aphasia^18–21^ and semantic dementia^22–24^. Notably, computational models can help simulate lesion effects by altering various aspects of the model’s cognitive architecture and processes and subsequently comparing the model’s performance with individual cases of impairment^25,26^, and have the potential to simulate recovery by retraining its computations affected by the simulated lesion.

Our prior work with the DISLEX computational model^27^ examined the feasibility of this approach and demonstrated preliminary support for accurate simulations of both (i) lexical access impairments in the native (L1) and the second language (L2) in Spanish-English BWA^28^ and (ii) response to therapy focused on word retrieval deficits regardless of the treatment language^29^. These previous simulations set the foundation for the current work, which aims to expand our computational modeling approach and validate its capacity to predict the optimal treatment language, leading to maximum therapy gains in both languages in BWA with varying prestroke bilingual backgrounds and varying L1 and L2 impairment profiles^30^.

As a first step towards this overarching goal, we developed BiLex^31^, a neural network model based on Self-Organizing Maps (SOMs)^32^, that accurately simulates L1 and L2 lexical access in picture naming in Spanish-English healthy bilinguals, while accounting for individual differences in their bilingual language learning history. Importantly, a cross-validation procedure demonstrated that the simulation capacity of BiLex generalized to predict L1 and L2 lexical access performance in other participants excluded from model training, thus validating its use to simulate prestroke naming ability in Spanish-English BWA.

The present study reports on the next step of this approach: the development and cross-validation of BiLex as a model that captures treatment outcomes in the treated and the untreated language in BWA while accounting for factors known to determine post-stroke language impairment and recovery^33^. These factors include demographics (i.e., age), prestroke bilingual background (i.e., L2 age of acquisition, prestroke lifetime language exposure and use of each language), poststroke language use, and language impairment (i.e., poststroke semantic processing and picture naming performance). To this aim, we employed a retrospective dataset of Spanish-English BWA who had received therapy in one or the other language, and conducted three experimental simulations: (i) modelling prestroke L1 and L2 naming ability for each BWA (ii), modelling their L1 and L2 poststroke impairment through damage to the semantic and phonetic subsystems of the neural network model, and (iii) retraining the neural network model to simulate the effects of treatment in either language (e.g.: English) in both the treated (e.g.: English) and the untreated language (e.g.: Spanish).

## Methods

### Model architecture

**Figure 1:**
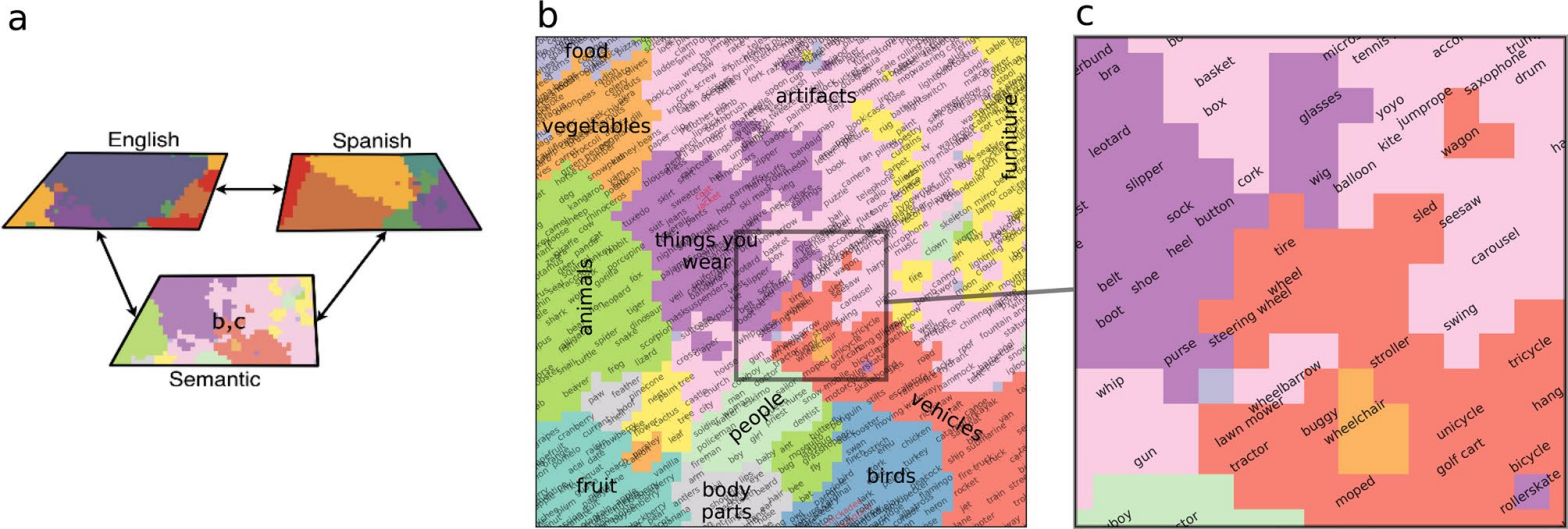
(**a**) The BiLex model consists of three interconnected SOMs, one for word meanings shared across languages, and two for their phonetic representations in L1 and L2. Bidirectional associative connections transfer activation between maps. (**b**) The semantic map organizes the model’s vocabulary according to word meanings, such that similar words are close together on the map. Plot **c** shows a detail of this map. Phonetic maps are organized in the same way, but reflect phonetic similarity. Created using Matplotlib v3.2.1 (https://matplotlib.org)^35^.

Following theoretical accounts of the bilingual mental lexicon^34^, the BiLex model is composed of a single shared semantic system and separate components for storing phonetic representations in the two languages. Each of the three components is implemented as a SOM^32^, and all three maps are connected via bidirectional associative connections that vary in strength depending on relative language dominance (Fig. 1).

BiLex is trained using feature-based semantic representations for 638 concrete nouns, along with their phonetic representations in English and Spanish. A standard SOM algorithm is used to train two maps at a time (e.g. training semantic and Spanish phonetic maps corresponds to exposure to a word in Spanish). Associative connections between the semantic and trained phonetic map are adjusted at the same time, increasing the connection strength between co-activated neurons using Hebbian learning. Additionally, activation flows from the semantic to the untrained phonetic map through associative connections, and is then used to train connections between phonetic maps. By varying the proportion of English vs. Spanish words trained over time, the model can approximate individual bilingual background profiles, capturing how lexical access in both languages varies with past language exposure^31^. The present study used the same modeling procedure to create a prestroke BiLex model for each BWA based on the patient’s past bilingual exposure, serving as the basis for individual simulations of stroke damage and treatment effects.

### Participants

A retrospective dataset of 13 Spanish-English bilinguals with post-stroke aphasia (eight females, mean age = $56.31 \pm 17.72$ years; mean time post-onset = $3.61 \pm 4.91$ years) was employed to optimize and validate BiLex treatment simulations. All were native Spanish speakers, reported normal or corrected-to-normal vision and hearing, and no history of psychiatric or neurological illness other than stroke (Table 1). All research was performed following relevant guidelines approved by the Boston University Charles River Campus Institutional Review Board, and all patients provided informed consent. All patients completed a Language Use Questionnaire (LUQ)^36^, providing detailed information on their individual history of language exposure, as well as comprehensive language testing^37^, including the Boston Naming Test (BNT)^38,39^ to assess picture naming ability in each language, and the Pyramids and Palm Trees test (PAPT)^40^ to evaluate language-independent semantic knowledge.

**Table 1:** Demographics, bilingual background, language scores and treatment information that served as input for simulations of language impairment and treatment outcomes. *AoA* age of acquisition, *Span* Spanish, *Eng* English, *Lang* Language, *BNT* Boston naming test, *PAPT* Pyramid and Palm Trees Test.

Patient ID	Years post onset	Age at testing	English AoA	% Lifetime exposure Span/Eng	% Poststroke lang. use Span/Eng	BNT Span/Eng (60 max)	PAPT (52 max)	Treated lang.	No. of treatment sessions
P1	7	44	19	72/28	78/22	26/22	48	Eng	10
P2	14	37	7.5	26/74	34/66	7/25	49	Span	13
P3	1	66	45	87/13	98/2	9/0	27	Eng	17
P4	0	34	12	71/29	54/46	0/0	52	Eng	10
P5	6	55	0	25/75	6/94	1/0	43	Eng	18
P6	13	54	21	55/45	50/50	27/28	47	Span	9
P7	1	56	5	37/63	42/58	11/25	48	Span	10
P8	1	54	6	34/66	45/55	4/31	45	Eng	7
P9	2	73	17	66/34	100/0	19/17	40	Span	10
P10	1	86	69	95/5	70/30	0/0	37	Span	10
P11	1	89	5	28/72	1/99	0/1	25	Eng	10
P12	0	42	18	90/10	71/29	28/3	43	Span	10
P13	0	42	9	68/32	71/29	1/0	47	Span	10

### Language treatment

Patients received a semantic feature analysis-based treatment for word-retrieval deficits^41^ in either English or Spanish (7–18 treatment sessions). Therapy involved training different types of semantic features (e.g.: “is sweet”, “can fly”) for target words, as well as training word retrieval in the target language. Naming accuracy on treatment words was tested in both languages preceding each treatment session, and treatment was discontinued when naming accuracy in the treated language either exceeded 80% or showed no significant improvement for three successive sessions. The results of naming probes before, during, and after treatment were used as target data for treatment simulations.

Patients were treated on 10–17 individually selected words that they previously failed to name in both languages. In cases where not enough such words were available, words the patient failed to name in only one language were used, leading to initial naming scores on the treatment set that were not always zero. To ensure that patients’ symptoms were stable before the start of treatment, these baseline scores were determined by averaging three to five naming probes in both languages.

### Simulating lexical access in BiLex

Lexical access in BiLex is simulated by presenting the semantic map with a semantic representation of a word (a binary vector encoding semantic features). The resulting map activation is transferred to the target phonetic map, and a word is counted as correctly named if the most highly activated phonetic unit is associated with the same input word. The naming accuracy of the model on a set of words is the proportion of words correctly named in this way.

The PAPT^40^ is a test of semantic processing that consists of 52 trials of word triplets. It requires selecting between two options (e.g.: a palm tree and a fir tree), the concept that is more closely related to the target (e.g.: a pyramid). For the BiLex semantic simulation, triplets included two word options (e.g.,“hood”, “pants”) that were similar (e.g.: things to wear) but differed in one defining feature (e.g.: worn on the head) shared with the target word (e.g.: “hat”) which could be used to determine the correct choice. To simulate each test trial, all three words were presented to the semantic map. The feature vectors of the winner neurons were used to determine the distinguishing feature (worn on head), and the map representation of the first word was then used to determine the correct choice. Fully trained and undamaged BiLex models scored about 96% on the simulated test, consistent with human data^40^.

### Simulating stroke damage and language impairment

**Figure 2:**
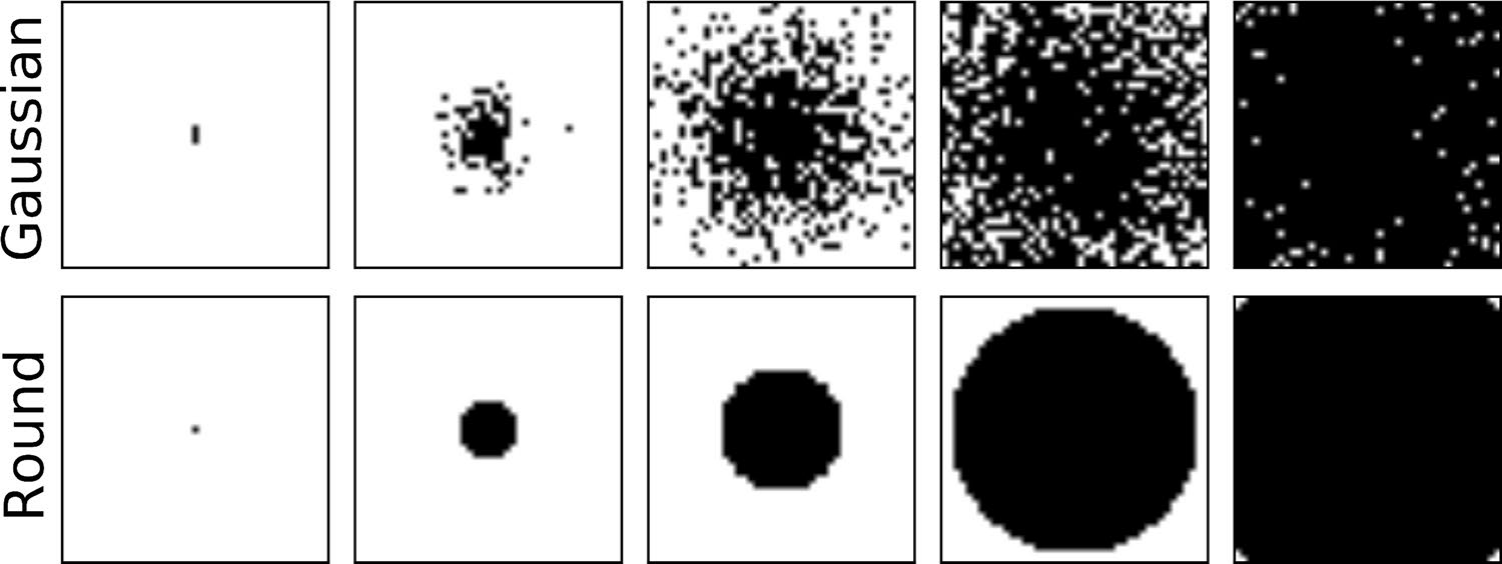
Two different methods to damage BiLex’s maps in order to simulate stroke damage. Black pixels represent SOM neurons that are removed (along with their associative connections) and white ones those that remain intact. Moving left to right, the amount of damage increases from almost zero to nearly total; adjusting the damage in this way, impairment in real patients can be matched.

Post-stroke models for each patient were created by training individual BiLex models up to the age of stroke onset, and then systematically lesioning (i.e., damaging) each model to match the patient’s impairment profile. Stroke-induced damage was simulated by deleting neurons along with their associative connections from the semantic and phonetic maps. Different lesion patterns for the model’s maps were evaluated (Fig. 2). In preliminary experiments, damaging a circular map area (bottom row) led to higher-quality treatment models; this type of lesion damage was therefore used in the rest of the experiments.

**Figure 3:**
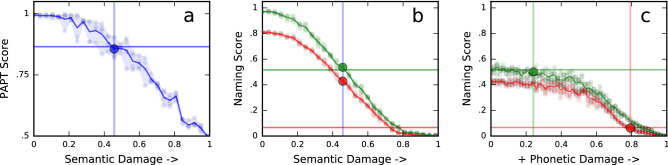
Adjusting the amount of damage to BiLex’s semantic and phonetic maps to match patients’ test scores (data for patient P8 are shown). (**a**) As lesion damage to the semantic map increases (left to right), the model’s semantic performance (PAPT score) decreases. The vertical line shows the amount of damage that best matches the target (horizontal line). (**b**) Semantic damage affects naming performance in both English (green) and Spanish (red). (**c**) Starting from the best-fit amount of semantic damage, additional lesion damage is applied to English and Spanish maps to match naming performance in both languages. As before, horizontal lines are the patient’s actual scores, and vertical lines are the best-fit amounts of lesion damage. The final lesion levels for semantic, English, and Spanish maps combine to reproduce the patient’s diagnostic scores in the model.

Increasing levels of damage were applied to the semantic map until the simulated PAPT score matched the patient’s score as closely as possible. Figure 3a illustrates this process for a specific patient model (P8). Figure 3b shows how semantic damage affects the model’s naming scores in both languages. In most cases, the amount of damage that reproduced the PAPT score did not by itself explain the target naming impairment. Thus, additional damage was applied to each model’s phonetic maps until naming performance matched that patient’s in both languages (Fig. 3c).

For many patients, significant time passed between stroke onset and treatment (up to 14 years; Table 1). The lesioned models were therefore trained further after lesioning, and the fit of PAPT and naming scores was evaluated when the models matched the patient’s age at treatment.

### Treatment simulations

To simulate the effects of treatment observed in each patient, post-stroke models were retrained on a subset of 30 words, individually chosen from the entire training corpus. Treatment words for each patient model were selected randomly, but matching the patient’s average baseline probe score. The treatment simulation itself was designed to mirror the actual treatment protocol used for patients:

1. In order to replicate the semantic feature-based portion of treatment, the model’s semantic map was retrained using the 30 treatment words as inputs.
2. To simulate repeated exposure to the treatment words in the treated language, the treated-language phonetic map, the semantic map, and the associative connections between them were retrained on the set of treatment words.
3. To give the BiLex model an opportunity to recover correct word associations in the untreated language,
  - Activation from the treated-language phonetic map was transferred to the untreated map through associative connections, and was then used to retrain connections between semantic and untreated phonetic maps.
  - Activation from the semantic map was transferred to the untreated map through associative connections, and was then used to retrain connections between the two phonetic maps.

After each retraining cycle, simulated naming performance was tested in each language to measure single-session effects in both languages that could be then compared with the actual treatment effects for each patient. Additionally, to account for the effects of language exposure over the course of treatment, the model was trained between treatment sessions for the equivalent of one week.

Each component of the treatment simulation was governed by parameters adjusting the intensity of retraining for SOMs and associative connections (i.e., learning rates). Additionally, Steps 3a and 3b could be applied conditionally, i.e. (i) only for words that were correctly named in the treatment language, (ii) only for words that the lesioned model can correctly translate between languages, or (iii) for all treatment words. Overall, a set of six parameters needed to be adjusted, including four parameters encoding learning rates, and two selecting the conditions for steps 3a and 3b.

An evolutionary algorithm (EA)^42^ was used to find parameter settings that optimally fit the treatment response across the training set of available patients. EAs are optimization algorithms inspired by biological evolution, that maintain a population of candidate solutions to a specific problem. By successively selecting higher-quality candidates and recombining them to form the next generation, EAs are often able to find near-optimal solutions to large nonlinear optimization problems such as the present one. The population consisted of 30 parameter sets, initially chosen at random. Each candidate was used to run a treatment simulation for all patients, using the individual poststroke BiLex models as a starting point. To evaluate the fitness of a candidate *C*, the model’s error was computed as the mean-squared difference between simulation and patient data across both languages and all patients in the training set *T*:

$$\begin{aligned} {\text {err}}(C) = \frac{1}{|T|} \sum \limits _{i \in T} \frac{1}{n_i}\sum \limits _{j=0}^{n_i}{((E_{ij} - \hat{E}^c_{ij})^2 + (S_{ij} - \hat{S}^c_{ij})^2)}, \end{aligned}$$

where $n_i$ is the number of treatment sessions for patient *i*, $E_{ij}$ and $S_{ij}$ are the English and Spanish naming scores for patient *i* after *j* treatment sessions, and $\hat{E}^c_{ij}$ and $\hat{S}^c_{ij}$ are the model’s estimates for these scores, using the parameter set *C*.

Based on current-generation errors, high-quality models were selected, and then recombined and mutated (subjected to a small amount of random noise) to form the next generation candidates. Evolution continued in this way for 100 generations, but restarting from a new random population if no new best-fit candidate was found for 20 successive generations.

To evaluate the accuracy of predicted treatment responses, a leave-one-out cross-validation experiment was conducted, with a different patient left out of the training set *T* for each separate EA optimization run. Each time, the treatment models that fit the training set best were then used to predict the treatment response for the left out patient, ensuring that predictions were based on previously unseen patient data.

## Results

### Actual and simulated language impairment

**Figure 4:**
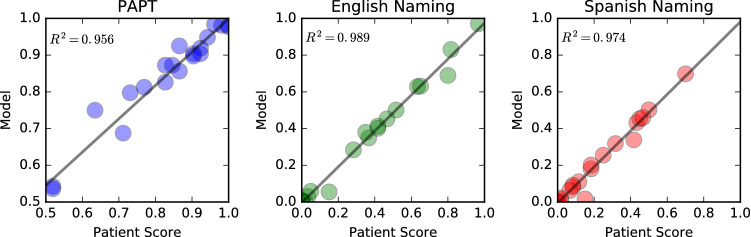
Individual pre-stroke models for each patient are lesioned to match diagnostic scores as accurately as possible. Real vs. simulated scores for all 13 patients are shown for the PAPT test (left), and English (middle) and Spanish (right) picture naming. The resulting models are used as a starting point for treatment simulations.

Figure 4 shows real vs. simulated poststroke performance for all patients on the PAPT (N=13; $R^2=0.956$; $p \ll 0.0001$), as well as English ($N=13$; $R^2=0.974$; $p \ll 0.0001$) and Spanish ($N=13$; $R^2=0.974$; $p \ll 0.0001$) naming. These results indicate that lesioned patient models were able to match poststroke semantic impairment, as well as naming impairment in both languages reliably. In patients with semantic deficits, damage to the semantic map alone affected both semantic and naming performance, but damage to all three maps was required in most patients (10/13). Importantly, damage to the semantic map alone never led to naming impairments that were more severe than those seen in patients, suggesting that the damage model is indeed able to capture how underlying lesion damage leads to the impairment patterns seen in patients.

### Actual and simulated treatment outcomes in the treated and the untreated language

**Figure 5:**
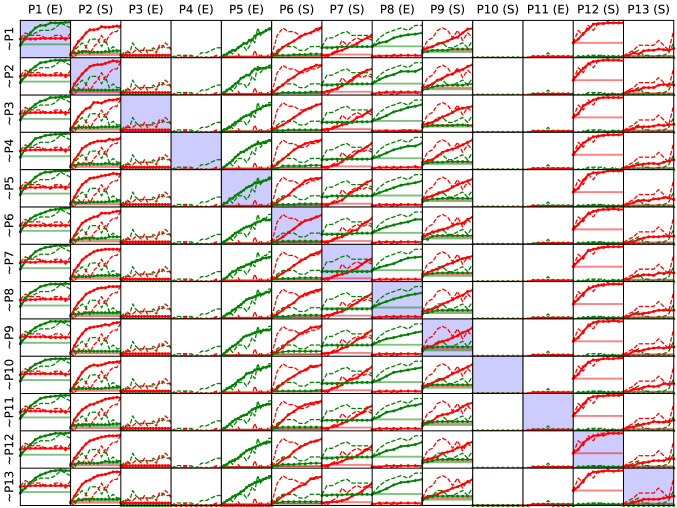
Leave-one-out cross-validation of the BiLex treatment model. Individual plots compare simulated (solid lines) and actual (dashed lines) patient treatment responses in English (green) and Spanish (red). Each row shares the same treatment model, optimized to fit all but one patient. Columns show the responses for each patient, with the treated language (*E* English, *S* Spanish) indicated in the title. Similarity between on- and off-diagonal entries illustrates the model’s ability to predict the treatment responses of previously unseen patients.

**Figure 6:**
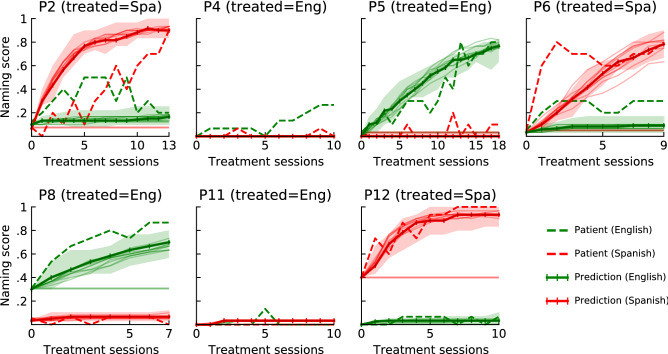
Robustness of the simulated results. For a representative subset of patients from the diagonal of Fig. 5, model variation is illustrated in two ways. First, the five best BiLex treatment models found by the evolutionary algorithm were identified based on their prediction accuracy on the training set; the median predictions of these models are shown in thin solid lines. Second, the minimum/maximum ranges over ten treatment simulations of the most accurate model are shown by the shaded area. There is very little variation across models and within the best model, demonstrating that the modeling approach is robust.

Figure 5 shows simulated responses for the best-fit treatment models, with a different patient left out in each row. The plots on the diagonal show predictions for patients that were not part of the training set. Figure 6 shows a more detailed view for a representative subset of patients on the diagonal. Each simulation was run 10 times; median predictions are shown in both figures, with shaded areas denoting the range in Fig. 6. In sum, the simulations were able to (i) capture treatment effects in the treated language over time in most cases (P1, P2, P5, P7, P8, P12) and at the end of treatment in others (P6, P9, P13); (ii) capture treatment effects in the untreated language both when the effects were large (P2, P7, P9) and when they were small (P3, P6, P13), (iii) demonstrate little effect for patients with a severe aphasia (P10, P11), and (iv) generalize these effects to previously unseen patients.

More specifically, to evaluate the quality of fit to the target data across all patients and treatment sessions, a mixed-effects model analysis was conducted, with patient treatment response in the treated language as the independent variable, predicted response and number of sessions as fixed effects, and patient as a repeated measure ($N=157$, 13 groups). For the treated language, the mixed model revealed significant dependence on predicted response ($p \ll 0.0001$), as well as the number of sessions received ($p \ll 0.0001$). The mixed model was superior to another using number of sessions received and baseline score as fixed effects ($p \ll 0.0001$), demonstrating that BiLex is able to capture treated-language effects in patients.

The same analysis was conducted for the untreated language, with one patient (P7) excluded because pre-treatment baseline probes showed consistent improvement (3-5-6 items out of 15 correctly named) in the untreated language, indicating that cross-language treatment effects could not be reliably separated from recovery unrelated to treatment. The mixed model ($N=146$, 12 groups) revealed that despite low cross-language patient responses overall, simulations were nevertheless predictive of actual outcomes. The mixed model was superior to one using baseline scores instead of model predictions ($p \ll 0.0001$). Further, predicted responses were accurate for seven patients showing low or no cross-language effect, and at least some cross-language effect was predicted in four of the remaining five cases. Thus, the results suggest that cross-language simulations may be useful in predicting whether the untreated language is likely to improve or not.

**Figure 7:**
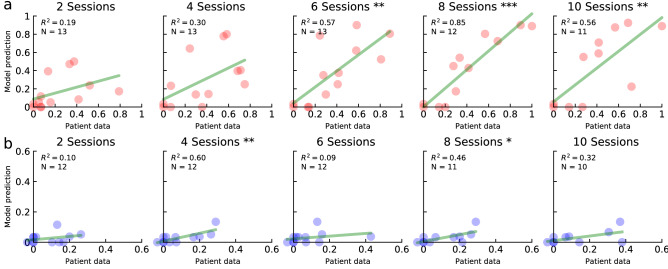
Regression models of treatment response for all patients at different times during treatment. As treatment progresses from left to right, model predictions (*y*-axis) become increasingly predictive of patient data (*x*-axis) in the treated (**a**) and untreated (**b**) language at different significance levels (*$p \le 0.05$; **$p \le 0.01$, ***$p \le 0.001$).

Simple regression analyses were conducted to evaluate whether simulated treatment response predicted actual patient treatment outcomes over the first 10 treatment sessions, for which there was sufficient patient data. Figure 7 shows actual versus model responses for all 13 patients. After four treatment sessions, the models predicted patient response in the treated language with significant accuracy, with $R^2$ values ranging between 0.54 and 0.82 (Fig. 7a). While still predictive, the models were less accurate for the untreated language (Fig. 7b, again excluding P7), generally underestimating the effects of cross-language transfer. Some possible reasons are discussed in the next section.

The best-fit discovered parameters for each of the mechanism underlying the treatment model are provided in Supplementary Table S1 online, and are further discussed in the supplement.

## Discussion

The present study employed the BiLex computational model^31^ to simulate language therapy effects in Spanish-English BWA. The patients’ prestroke naming ability was modeled in each language, a lesion component was implemented to imitate the effects of brain insult on the bilingual language system, and the lesioned models were retrained to simulate individual responses to language treatment provided in either language. The results indicate that BiLex can simulate therapy outcomes in BWA, achieving accurate and robust predictions of treatment effects in the treated language, while partially capturing different degrees of cross-language generalization effects in the untreated language. The cross-validation approach demonstrated that the EA is successful in optimizing training parameters for individual BiLex patient models. The resulting models predicted treatment effects accurately, and generalized to patients whose data were not used in model training. These findings highlight the potential of BiLex to capture treatment response in patients with different profiles of bilingualism and language impairment, making it possible to cope with the large inter-individual variability often reported in behavioral studies with small samples^3–5^.

The simulations of individual therapy outcomes in the treated language showed the closest approximation to actual patient performance on naming probes throughout treatment, with nearly identical simulated and actual treatment response curves for most patients (P1, P2, P5, P7, P8, P12). The models for other patients reflected final treatment outcomes, but were less accurate in predicting initial response to therapy (P6, P9, P13). In these cases, the model did not always fully capture the trajectory of change in naming ability during treatment, possibly reflecting intra-subject variability in lexical access often observed in aphasia due to post-stroke cognitive slowing and aphasia severity, word lexical properties, and lexical competition in the same language^43^ and across languages^44^. Overall, the results suggest that the BiLex model can successfully simulate language improvements in the treated language and capture central factors that modulate individual response to language therapy in BWA, including prestroke bilingual language learning history and poststroke impairment as suggested by bilingual aphasia research^45^.

Several patients did not show significant cross-language generalization, and these results were resonant with the corresponding computational predictions, thereby reflecting overall treatment benefits in the treated language only^37,46,47^. The findings in patients with significant cross-language effects are less conclusive. Simulations do predict such effects in four out of six such patients, suggesting that the involved mechanisms were at least partially captured by the model. Overall, however, the model tends to underestimate the effect. A likely reason is that the amount of available patient data used to train the EA was insufficient to fully characterize cross-language generalization. In line with prior studies showing that not all BWA show cross-language generalization effects^3–5^, only three patients in the present study showed significant cross-language therapy effects (P3, P6, P13), and three more patients showed isolated instances of improvement in the untreated language (P2, P7, P9). A second possible reason is architectural. The current model incorporated the training of cross-language associative connections between the phonetic maps of each patient model to reflect facilitation as a potential mechanism of cross-language transfer effects^37^. It is possible that alternative or additional mechanisms exist, including transfer through the semantic system^48^. Third, given the difference in magnitude and consistency between treatment effects in the treated versus the untreated language, it is possible that the evolutionary model-fitting procedure converged primarily on the much stronger signal of treated-language effects. Future research with BiLex will require larger patient datasets to improve simulations of cross-language transfer in bilingual aphasia and provide a more fine-grained characterization of the mechanisms that may account for these therapy effects.

Another important contribution of this work is the accurate simulation of the relative severity in the bilingual language system. Simulated PAPT and BNT scores strongly predicted patients’ actual language performance across different lesion approaches, capturing varying degrees of impairment in semantic processing and naming ability in each language. Moreover, by gradually adjusting the amounts of lesion damage in the semantic and phonetic maps, the model was able to capture qualitatively different profiles of (i) patients with minimal semantic impairment and predominant lexical access deficits and (ii) patients with both semantic and lexical deficits. These findings are in line with computational models that account for disruptions at different levels of word production in order to capture word retrieval deficits in monolinguals^18,23,24^ and bilinguals^28,29^, as well as behavioral evidence of both performance profiles in post-stroke aphasia^49,50^. The severity of language deficits has been strongly associated with early and long-term post-stroke aphasia outcomes^51,52^, and these effects are generally well-captured by the model. Importantly, the model’s ability to accurately capture varying degrees of severity and impairment was reflected in the absence of improvement in patients with severe language dysfunction (P10, P11). Only in two cases (P3, P4), with low but relatively stable response to treatment, the patient models predicted no treatment response. In these cases, the simulated lesions that best matched the impairments required deleting almost all neurons from either the semantic or the treated phonetic map, thus preventing any recovery in the model. Future versions of BiLex, with larger maps and vocabulary sizes, will likely make more fine-grained damage models possible.

Although the architecture of BiLex and its main training algorithm are similar to the previous DISLEX model^29^, BiLex incorporates several technical advances. First, BiLex uses a larger corpus of 638 words with much more detailed semantic and phonetic presentations for SOM training. Second, the current prestroke patient models are based on healthy bilingual performance on standardized language tests as well as fine-grained data on factors that determine prestroke bilingual profiles. They share the same EA-optimized training schedule, leading to simulations that can predict naming performance in healthy bilinguals across different ages and profiles of bilingualism more accurately than before^31^. Third, post-stroke models employ more fine-grained lesion mechanisms that distinguish semantic impairment from phonetic damage. Fourth, treatment simulation is governed by a set of training parameters optimized to match human treatment effects. These improvements relative to the prior DISLEX model enable BiLex to produce more principled and accurate simulations of impairment and treatment effects.

These advances also lead to promising avenues of future research. For instance, a version of BiLex trained on all 13 patients is currently used to make a priori predictions about the optimal treatment language for BWA in a randomized controlled trial^30^. In this study, the optimal treatment language is defined for each patient by comparing simulated effects in both languages when treatment is provided in one versus the other language. Experimental patients receive the model-prescribed optimal language of treatment while control patients receive therapy in the model non-prescribed language, and the goal is to validate BiLex predictions by contrasting simulated versus actual treatment outcomes in patients receiving therapy in the language defined as optimal by the model and patients receiving therapy in the language opposite to the model’s recommendation. Also, future versions of BiLex could incorporate additional parameters to account for other factors that may influence treatment outcomes in BWA, including lesion location^45^, deficits in cognitive control^53^, and patterns of language switching and mixing^6,54^, as well as therapy-related factors such as type of therapy, duration, and intensity^52^. Finally, inconsistencies in naming ability at the individual item level are common in aphasia and could play an important role in characterizing patterns of impairment and recovery. Future work with BiLex could account for within-subject fluctuations in naming performance and different types of errors at the individual patient and item level.

In conclusion, BiLex provides a useful framework to understand different sources of language deficits in BWA and to generate predictions of treatment outcomes, accounting for relevant factors that modulate treatment-induced recovery in aphasia. In the future, improved BiLex patient models could be used to inform therapy and tailor individual treatment schedules to the patient’s unique bilingual profile, deficits and communication needs.

## Supplementary Information

Supplementary Information.

## Data Availability

The demographic, clinical, and simulation datasets generated for this study are available upon request.
