# Supplementary material for: Predicting language treatment response in bilingual aphasia using neural network-based patient models
Source: Sci Rep. 2021 May 18;11:10497. doi: 10.1038/s41598-021-89443-6 (PMC8131385; doi:10.1038/s41598-021-89443-6)
Supplement: Supplementary file 1 — Supplementary Information. [file 41598_2021_89443_MOESM1_ESM.pdf]

Predicting language treatment response in bilingual aphasia using neural network-based patient models

Uli Grasemann[1+*], Claudia Peñaloza[2+*], Maria Dekhtyar[2], Risto Miikkulainen[1], and Swathi Kiran[2]

[1]Department of Computer Science, The University of Texas at Austin, Austin, TX, 78712, USA
[2]Department of Speech, Language and Hearing Sciences, Boston University, Boston, MA, 02215, USA
[*]uli@cs.utexas.edu; penaloza@bu.edu
[+]These authors contributed equally to this work

Supplement - Discovered Treatment Parameters

Table S1 shows discovered best-fit parameters for each mechanism underlying the treatment model and each training set (i.e. each left-out patient). Mechanism numbers refer to those used in the "Treatment Simulations" section. Evolved parameters governing learning rates are expressed as multiples of the learning rates used during regular model training; actual learning rates used in treatment simulations thus depend on (i) whether SOMs or associative connections are trained, and (ii) the age of the patient at the time of treatment.

Patient Left Out	Mechanism 1 Learning rate	Mechanism 2 Learning rate	Mechanism 3a Learning rate	Mechanism 3a Condition	Mechanism 3b Learning rate	Mechanism 3b Condition
P1	2.201	2.483	2.637	Trans	0.939	Named
P2	4.401	2.850	2.379	Trans	4.536	Named
P3	4.558	2.197	3.949	Trans	0.867	Trans
P4	4.163	2.568	2.393	Trans	3.782	Named
P5	3.545	2.860	2.783	Trans	4.632	Always
P6	3.024	2.075	1.668	Trans	0.000	Named
P7	3.877	2.638	3.936	Trans	2.843	Named
P8	0.463	2.173	3.201	Trans	4.369	Named
P9	1.710	1.694	4.165	Trans	1.213	Always
P10	1.621	2.689	2.691	Trans	1.512	Always
P11	1.812	2.297	4.323	Trans	1.106	Trans
P12	2.927	2.401	3.490	Trans	3.817	Trans
P13	1.539	2.276	2.614	Trans	3.072	Always

Table S1. Discovered best-fit parameters for each patient and each mechanism underlying the treatment model. "Patient Left Out" indicates the patient excluded from the training set. Learning rates are expressed as multiples of those applied during regular (prestroke) model training. Mechanisms 3a and 3b are applied conditionally: "Trans" = Applied only for correctly translated words; "Named" = Applied for words named correctly in the trained language; "Always" = Applied for all words in the treatment set.

The evolved parameters suggest possible conclusions about the explored parameter space and the treatment model. For example, the learning rates for Mechanism 2 (mean=2.4, SD=0.33) are less variable that those for Mechanism 1 (mean=2.76, SD=1.30) across training sets, possibly indicating that Mechanism 2 may have a more central role in modeling recovery in real patients. However, it is important to note that the training sets for these discovered parameters overlap significantly, so unlike in the case of predicted treatment responses, results are not independent, and such conclusions should therefore be considered very carefully. Moreover, the high consistency of simulated treatment responses across EA runs (columns in Figure 5) suggests that rather than a single best parameter set, there may be interdependent ranges of highly predictive parameters. Future research with larger patient data sets will make it possible to characterize high-fitness treatment parameters more fully.
